# How does social integration work when older migrants obtain health services from community? Evidence from national database in China

**DOI:** 10.3389/fpubh.2023.1283891

**Published:** 2023-12-12

**Authors:** Shenshen Liu, Bo Qin, Dongyang Wang

**Affiliations:** ^1^Department of Disease Control and Prevention, General Hospital of Central Theater Command, Wuhan, China; ^2^Hubei Key Laboratory for Kidney Disease Pathogenesis and Intervention, Hubei Polytechnic University School of Medicine, Huangshi, China; ^3^Department of Nursing, The Third People's Hospital of Henan Province, Zhengzhou, China

**Keywords:** community-based, health services, Chinese older migrants, health status, social integration

## Abstract

**Background:**

The roles of community are often overlooked when studying the older migrants' health issues, and more importantly, the mediating effect of social integration on the health of older migrants were rarely investigated empirically.

**Methods:**

This study developed comprehensive index to explore this relationship. With data from the 2017 China Migrants Dynamic Survey, the study first examined the potential linkage between community-based health services and the health of older migrants. Ordered logit regressions was carried to investigate whether the self-rated health of older migrants is related to health education and health records provided by community, then the Causal Stepwise Regression and bootstrap method was used to looked into the potential mediation effect.

**Results:**

The findings showed that older migrants with more community-based health education had higher self-rated health (β = 0.038, SE = 0.009, *p* < 0.001). However, the community-based health records were not associated with older migrants' health. Moreover, higher levels of social integration were associated with community health education (β = 0.142, SE = 0.014, *p* < 0.001), and social integration was positively associated with older migrants' health (β = 0.039, SE = 0.002, *p* = 0.024), indicating the mediation role of social integration.

**Conclusion:**

The vital role of community-based health education in improving the health of older migrants was found, and social integration plays a mediating role.

## 1 Introduction

The emergence of the migrant older adults in China is accompanied with urbanization and the large-scale rural-to-urban migration (1). Migrant older adults denote those over 60 years old who have departed from their household registration residence for a period exceeding 6 months, due to reasons such as working or providing care for grandchildren (2). Different from the western countries, in China, with extended family value, the older people also move alone with their children' migration. And it has been common for those older migrants to look after grandchildren (3). As migrant patterns become increasingly centered around family, the number of older migrant individuals has grown rapidly, becoming a crucial group for driving China's society and economy development (4). According to a survey conducted for the China Migrant Development Report 2018, the number of these people was approximately about 20 million, accounting for 8% of all migrant population (5). The scale would further increase in the future, along with the ongoing urbanization and population aging (6). The older migrants consequently become what should be focused.

The older migrants' health is a significant concern of the aging society and influences the function of family, however, these people usually face more vulnerability than other migrant people due to aging (7, 8). Firstly, their ability to adapt to the new environment in the host city is decreasing with aging (9). As the duration of residence in new environment increases, long-term exposure to the accumulation of chronic stress significantly reduces the health status of older migrants (10, 11). They actually need more health recourses than the younger and urban counterpart. However, since the distribution of urban health resources in China is based on household registration status, most of them are excluding from urban public healthcare services and other social welfare benefits (12, 13). Previous research has suggested a high percentage of chronic illnesses and a low ratio of hospitalization among them (14, 15). Secondly, the social capital of these migrant people has been limited when the leave their original place to an unfamiliar city (16). In the host city, the majority of them has no fixed workplace and other social organizations who could act as supporters, nor do they have diverse social activities. Thus, their social circle and interaction is limited to the community they live, showing a high dependency on the community (17). This increases the importance of community in providing health productions and services. However, to date, the health issues has been regarded as a family responsibility for a long term (18, 19), resulting in an ineffective health maintenance and improvement for these people.

Looking into community health services could provide a practical perspective on the wellbeing of migrants. Previous research on immigrants in Europe and America has confirmed that the enhancement of community-based healthcare services improved international migrants' health (20, 21). However, we could not know to what extent the same could be a case for China. To date, the health issues of older migrants in China mainly analyzed from individual characteristics and family characteristics of older migrants (22, 23). Among the very limited studies that took the community as an analysis unit, they have addressed the informal community services, supports and interactions, all of which roles have been proved as positive and significant in influencing older migrants' overall health (24, 25), the community-based formal service support was neglected. In addition, the deeper underlying mechanisms behind in the relationship between the community support and the older migrants' health are unclear (26). It is necessary to pay attention to especially the formal public health services provided by the community and explore their role in health. Not only in academics, but also in practice, the narrowed scope of health issues on individual and family prescriptive results in an ineffective health maintenance and improvement for these people. Actually, the Chinese central authorities have emphasized the importance of community development. 2019 National Medium and Long-term Plan for Actively Coping with Population Aging has encouraged communities to facilitate service provision for senior citizens, improve community-based public health education, enhance citizens' quality of life, and make substantial contributes toward optimal aging. However, as there are in effect almost no formal provision public health services for older migrants in communities, Chinese older migrants often lack a comprehensive understanding of community-based public services.

One potential associated mechanism in community-based services affecting the older migrant's health may be social integration. According to the social capital theory, community is an important platform for promoting and accumulating social capital for older immigrants, the formation of community social capital requires the community to transform the residents' interaction needs into actual interaction behaviors (27, 28). Previous studies have already demonstrated that the interactive activities in the community are a process of community social capital accumulation (29, 30), community can create such opportunities to interact with others as various health services includes offline health promotion and health education lecture, and the positive relationships formed can enhance the recognition of the older migrants by local residents, which can enhance the social integration of the migrant population (31). In addition, research has also found association social integration and the health outcomes of migrants (32, 33). Based on the Transform program, which is a poverty alleviation program in the Philippines, explored the mechanisms by which social capital at the community level affects self-rated health, and revealed that access to connections with others in the community through the program significantly enhanced self-rated health by 17% (34). Besides, the community support also can be viewed as a sociocultural and spiritual resource that ultimately contributes to improved overall health by strengthening their sense of community identity and alleviating personal stress and sense of loneliness, ultimately improve their overall health (35, 36).

Focusing on the understudied community context, this study aims to investigate the association between community-based health services and the health of older migrants in China, and further to explore the underlying mechanisms behind in the relationship. As the older migrant population is increasing rapidly in China, such analyses and findings provide insights for improving the health of this population and promoting specific behaviors related to community public health service provision.

## 2 Materials and methods

### 2.1 Study sample

This paper uses the 2017 China Migrants Dynamic Survey (CMDS), which was obtained from a survey conducted by the National Health and Family Planning Commission. The survey covers 31 provinces, autonomous regions and municipalities, the Xinjiang Production and Construction Corps in China, using the probability proportional scale sampling (PPS) method to survey the migrant population aged 15 years and older (37). All sample individuals are surveyed through face-to-face interviews and questionnaires. Besides, the CMDS data covers the questionnaires on public health services, migrant and residence intention and social integration, with broader range of variables and good reliability and validity, which can provide a data source for this study. Additionally, although the CMDS data from 2017 is survey data collected before the pandemic, it remains highly representative and consistent with the current trend of China's migrant population, making it applicable to the context of 2023. The 2017 CMDS data contained a total of 169,989 samples. Out of the total sample, a total of 5,986 older people over the aged 60 were extracted. Additionally, there were 646 samples with missing values in each variable and those who answered “don't know,” “not sure,” and “can't answer” options were eliminated, and finally 5,340 valid samples were obtained.

### 2.2 Measures

#### 2.2.1 Dependent variable

Self-rated health does not only reflect the subjective experiences of older migrants, composed of their daily health behaviors, psychological status and major illnesses, but also include their general feelings about socialization, which can comprehensively evaluate both subjective and objective aspects of individual health (38). In this paper, the questionnaire “How do you feel about your own health now” was selected to measure the health of older migrant people. The question includes four options: 1. healthy; 2. generally healthy; 3. unhealthy but able to take care of themselves; and 4. unable to take care of themselves. To facilitate the analysis and maintain consistency with the independent variables, the options were recoded as “very poor,” “poor,” “better,” and “very good,” respectively from 1 to 4. In addition, the four categorical variables of self-rated health in the original questionnaire were directly retained in order to ensure the accuracy of data (39).

#### 2.2.2 Independent variables

Community-based health services were measured through health education and health records, because these were the only two public health service programs that were available to the entire population in China, which implied that older migrant individuals have access to these services (40). Community health education was selected from the question “In the past year, did you receive health education in your current village/community”, with nine options including “occupational disease prevention and control,” “AIDS prevention,” “reproductive health,” “tuberculosis prevention and control,” “smoking prevention and control,” “mental health,” “chronic disease prevention and control,” “self-help in public emergencies,” and “eugenics and excellent pregnancy knowledge.” The nine questions were treated as dummy variables, with “yes” coded as “1” and “no” coded as “0”. On this basis, the total value of these nine variables was calculated to measure the overall level of community-based health education.

To determine the availability of community health records, we selected the question “Have you established a local health record?” with answer options of 1. “yes, established”; 2. “no, never heard of it”; 3. “no, but heard of it”; and 4. “not sure”. In order to reflect the availability of community health records directly, the variable was treated as a dichotomous variable after excluding those who answered “not sure”. Those who responded “yes, already established” were considered to be “providing community health records” and were coded as “1”. Those who responded “no, never heard of it” and “no, but heard of it” were considered as “providing community health records”. The responses “no, never heard of it” and “no, but heard of it” were considered as “not established” and were coded as “0”.

#### 2.2.3 Mediator variable

The mediator variable was social integration. Measured by the agreement with the statements: “I like the city/place where I live now,” “I am concerned about the changes in the city/place where I live now,” “I would love to integrate among the locals and become one of them,” “I feel that the locals are willing to accept me as one of them,” “I feel that I am already a local,” “I feel that locals look down on outsiders,” “It is more important for me to do things according to the customs of my hometown,” and “My hygiene habits are quite different from those of local citizens.” Each question includes four options: 1. fully disagree; 2. disagree; 3. basically agree; 4. fully agree. Drawing on relevant studies (41, 42), the scores of eight variables were combined into a total score using factor analysis. The score ranged from 8 to 32, with the higher the value taken, the higher the social integration level of the respondent.

#### 2.2.4 Control variables

According to existing studies, three categories of factors should be taken into account when studying older migrant populations including demographic, socioeconomic, and migrant characteristics. Individual characteristics variables include sex (0 = female; 1 = male), household registration (0 = urban; 1 = rural), marital status (0 = without spouse; 1 = with spouse), and education level (1 = primary school or less; 2 = junior high school; 3 = high school or more). Socioeconomic characteristics variables include personal income, measured by log of family income, work status (0 = without work; 1 = with work), and insured status (0 = no; 1 = yes). Migrant characteristics variables include migrant range (1 = interprovincial migrant; 2 = intercity migrant within the province; 3 = intercounty migrant within the city), migrant reason (1 = doing business and working; 2 = caring for children; 3 = migrant for aged-care; 4 = others), and the time of migrant.

### 2.3 Data analysis

The statistical analyses were conducted by STATA SE Version 15.1. Descriptive analysis was performed to investigate the initial differences in the sample, including the mean, standard deviation, minimum, and maximum values of all variables in the sample. Ordered logit regression was conducted to investigate the association between community-based health services and the health of older migrants. Model 1 examined the association between all control variables and self-rated health. Model 2 included community health education and examined their effect on self-rated health. Model 3 included community health records and examined their effect on self-rated health. Model 4 incorporated all variables to fully examine the association of community-based health services and other variables on health, as well as the resulting coefficient changes. The Causal Stepwise Regression (CSR) method was used to analyze the mediating effects of social integration by decomposing the total effect of community health services on the self-rated health of older migrants into direct and indirect effects. CSR is a commonly used method for identifying causality. By gradually adding independent and control variables (43), it can determine which variables have the significant impact on the health of older migrants. In addition, to test the robustness of the study results, a sensitivity analysis was performed by selecting the substitute variables of dependent variable. Substitute variables include disease status and general health index, in which general health index was an operational treatment of self-rated health, chronic disease and common disease indicators by means of average sum (44).

## 3 Results

### 3.1 Descriptive statistical analysis

Table 1 presents a comprehensive overview of the key characteristics of the sample of older migrant individuals. The mean value of self-rated health of older migrants was 3.220 (SD = 0.773; range = 1–4). In community-based health services, the mean value of community health education was 2.702 (SD = 3.197; range = 0–9), and only 33.4% of older migrants had community health records. The mean value of social integration was 25.88 (SD = 3.424; range = 8–32). Among the 5,340 older migrant respondents, more than half were male (58.09%). The majority of the older migrants had rural household registration (56.62%), and 84.19% of the older migrants married. On the education level of older migrants, 47.79% had the education level of primary school and below, 30.05% had education level of junior high school, and 22.16% had education level of high school or above. The mean score of the logarithm of family income was 8.332 CNY (SD = 0.914; range = 4.094–11.408). A minority of the sample had a job (29.15%), and most of the sample enrolled in health insurance (65.85%). The migrant range was mainly interprovincial migrant (44.86%), intercity migrant within the province (34.28%), and the lowest percentage was intercounty migrant within the city (20.86%). The most common reason for migrant is to take care of children and grandchildren (40.54%), then followed by migrant for doing working (34.88%), and migrant for aged-care in other places (13.57%), in addition to other reasons for migrant (11.03%). Finally, the mean value of migrant times was 9.616 (SD = 8.177; range = 0–70).

**Table 1:** Sample characteristics of the older migrants (*N* = 5,340).

**Variables**	**Mean/%**	**SD**	**Range**
**Dependent variables**			
Self-rated health	3.220	0.773	1–4
**Independent variables**			
**Community-based health services**			
Community health education	2.702	3.197	0–9
Community health records	0.334	0.472	0–1
**Mediator variable**			
Social integration	25.88	3.424	8–32
**Control variables**			
**Sex**			
Male	58.09%		
Female	41.91%		
**Household registration**			
Rural	56.62%		
Urban	43.38%		
**Marital status**			
With spouse	84.19%		
Without spouse	15.81%		
**Education level**			
Primary school or less	47.79%		
Junior high school	30.05%		
High school or more	22.16%		
Log of family income (CNY)	8.332	0.914	4.094–11.408
**Work status**			
With work	29.15%		
Without work	70.85%		
**Insured status**			
Yes	65.85%		
No	34.15%		
**Migrant range**			
Interprovincial migrant	44.86%		
Intercity migrant	34.28%		
Intercounty migrant	20.86%		
**Migrant reason**			
Migrant for work	34.88%		
Migrant for childcare	40.54%		
Migrant for aged-care	13.57%		
Other reasons	11.03%		
Migrant times	9.616	8.177	0–70

### 3.2 The associations between community-based health services and the health of older migrants

Table 2 displays the results of the regression analysis, revealing the correlation between community-based health services and the health status of the older migrant population. Notably, the results were consistent and uniform across all models, only having community-based health education were associated with higher self-rated health (β = 0.038, SE = 0.009, *p* < 0.001), and a negative correlation between community health records and older migrant' self-rated health (β = −0.091), but there was no statistical significance (SE = 0.059, *p* > 0.1). Additionally, other noteworthy factors that positively influenced their health included sex (β = 0.151, SE = 0.057, *p* < 0.001), completing junior high school education (β = 0.274, SE = 0.066, *p* < 0.001), high school education and above (β = 0.327, SE = 0.081, *p* < 0.001), higher family income (β = 0.354, SE = 0.034, *p* < 0.001), work status (β = 0.812, SE = 0.079, *p* < 0.001), interprovincial migrant (β = 0.259, SE = 0.072, *p* < 0.001), intercity migrant (β = 0.162, SE = 0.073, *p* = 0.026) and migrant for work (β = 0.383, SE = 0.103, *p* < 0.001). Other factors that had a negative effect on the health of older migrants included older having a rural household (β = −0.227, SE = 0.074, *p* < 0.001), and migrant times (β = −0.013, SE = 0.003, *p* < 0.001).

**Table 2:** Hierarchical regression models of community-based health services on older migrants (*N* = 5,340).

**Variables**	**Model 1**	**Model 2**	**Model 3**	**Model 4**

	β **(SE)**	β **(SE)**	β **(SE)**	β **(SE)**
Sex	0.136^**^	0.151^***^	0.148^***^	0.151^***^
	(0.056)	(0.057)	(0.057)	(0.057)
Household registration type	−0.221^***^	−0.220^***^	−0.234^***^	−0.227^***^
	(0.072)	(0.074)	(0.074)	(0.074)
Marital status	−0.046	−0.061	−0.055	−0.057
	(0.073)	(0.074)	(0.074)	(0.074)
Junior high school	0.286^***^	0.276^***^	0.285^***^	0.274^***^
	(0.065)	(0.066)	(0.066)	(0.066)
High school or more	0.331^***^	0.329^***^	0.342^***^	0.327^***^
	(0.079)	(0.081)	(0.081)	(0.081)
Log of family income (CNY)	0.373^***^	0.358^***^	0.364^***^	0.354^***^
	(0.033)	(0.034)	(0.034)	(0.034)
Work status	0.829^***^	0.815^***^	0.822^***^	0.812^***^
	(0.078)	(0.079)	(0.079)	(0.079)
Insured status	−0.002	−0.009	−0.006	−0.007
	(0.069)	(0.071)	(0.071)	(0.071)
Interprovincial migrant	0.254^***^	0.269^***^	0.245^***^	0.259^***^
	(0.070)	(0.072)	(0.072)	(0.072)
Intercity migrant	0.165^**^	0.164^**^	0.153^**^	0.162^**^
	(0.072)	(0.073)	(0.073)	(0.073)
Migrant for work	0.381^***^	0.387^***^	0.384^***^	0.383^***^
	(0.101)	(0.103)	(0.103)	(0.103)
Migrant for childcare	0.112	0.136	0.114	0.136
	(0.090)	(0.091)	(0.091)	(0.091)
Migrant for aged-care	0.066	0.078	0.074	0.080
	(0.105)	(0.107)	(0.107)	(0.107)
Migrant times	−0.013^***^	−0.013^***^	−0.013^***^	−0.013^***^
	(0.003)	(0.003)	(0.003)	(0.003)
Community health education		0.034^***^		0.038^***^
		(0.008)		(0.009)
Community health records			−0.013	−0.091
			(0.057)	(0.059)

β, estimated coefficient; Standard errors in parentheses; ^**^p < 0.05, ^***^p < 0.001. Model 1 denotes the regression results between each control variables and self-rated health. Model 2 denotes the regression results between community health education and self-rated health when all control variables were included. Model 3 denotes the regression results between community health records and self-rated health when all control variables were included. Model 4 denotes the regression results between community-based health services and self-rated health when both community health education and community health records were included as independent variables.

### 3.3 The mediating effect of social integration between community-based health services and older migrants' health

Table 3 presents the mediating role of social integration of older migrants in the relationship between community-based health education and self-rated health. The results showed a notable correlation between community health education and social integration among older migrant individuals (β = 0.142, SE = 0.014, *p* < 0.001), and social integration also had a significant positive effect on self-rated health of older migrants (β = 0.039, SE = 0.002, *p* = 0.024). Social integration played a vital mediating role in the correlation between community health education and the health status of older migrant individuals [indirect = 0.006, SE = 0.000, *p* < 0.001, 95% CI: (0.003, 0.008)], with the mediating effect accounted for 17.64% of the total effect.

**Table 3:** Mediating effect of society integration in the association between community-based health services on older migrants (*N* = 5,340).

**Variables**	**β**	**SE**	**Bootstrap 95% lower bound**	**Bootstrap 95% upper bound**	**% of total effect**
a coefficient	0.142^***^	0.014	—	—	—
b coefficient	0.039^**^	0.002	—	—	—
Indirect effect	0.006^***^	0.000	0.003	0.008	17.64%
Direct effect	0.028^***^	0.003	0.021	0.035	82.36%
Total effect	0.034^***^	0.008	—	—	—

β, estimated coefficient; SE, standard error. ^**^p < 0.05, ^***^p < 0.001; a coefficient denotes the regression results between community health education and social integration; b coefficient denotes social integration and self-rated health.

### 3.4 Sensitivity analysis

Table 4 presents the sensitivity analysis of association between community health services and older migrants' health. In the results that the relationship between community health services and disease status of the older migrants (Model 5), the direction of the coefficient opposite between sensitivity analysis and benchmarking analysis, and the size and significance of the coefficients were almost similar. Community health education was associated with lower probability of suffering from disease (β = −0.022, SE = 0.010, *p* = 0.028). This was essentially consistent with the benchmarking analysis, which community health education were conducive to older migrants' health.

**Table 4:** Sensitivity analysis for benchmarking analysis (*N* = 5,340).

**Variables**	**Model 5**		**Model 6**	
	**β**	**SE**	**β**	**SE**
Sex	−0.075	0.064	0.072^**^	0.031
Household registration type	−0.183^**^	0.081	−0.020	0.040
Marital status	0.193^**^	0.084	−0.087^**^	0.040
Junior high school	0.008	0.074	0.111^***^	0.036
High school or more	−0.093	0.090	0.189^***^	0.044
Log of family income	−0.069^*^	0.037	0.131^***^	0.018
Work status	−0.452^***^	0.088	0.390^***^	0.042
Insured status	0.006	0.078	−0.051	0.038
Interprovincial migrant	−0.068	0.080	0.131^***^	0.039
Intercity migrant	−0.080	0.081	0.057	0.039
Migrant for work	−0.340^***^	0.113	0.176^***^	0.056
Migrant for childcare	−0.044	0.100	−0.030	0.050
Migrant for aged-care	0.038	0.116	0.001	0.058
Migrant times	0.016^***^	0.004	−0.009^***^	0.002
Community health education	−0.022^**^	0.010	0.016^***^	0.005
Community health records	0.055	0.065	−0.007	0.032

In model 5, the dependent variable was disease status. In model 6, the dependent variable was general health index.

β, estimated coefficient.

^*^p < 0.1, ^**^p < 0.05, ^***^p < 0.001.

In the results that the association between community health services and the general health of the older migrants (Model 6), the coefficients of sensitivity analysis and benchmarking analysis were in the same direction. Providing community health education was associated with higher overall health level (β = 0.016, SE = 0.005, *p* < 0.001). Similarly, the coefficient of the relationship between community health records and older migrants' general health was negative value (β = −0.007), and the significance of coefficients were the same as the baseline regression (SE = 0.032, *p* > 0.1).

### 3.5 Endogenous test

There may be an endogenous relationship between the community-based health services and the health of older migrants. The instrumental variable method was employed to address the endogenous problem. An effective instrumental variable must satisfy two conditions. The first condition is correlation, where the instrumental variable is related to the community health services received by older migrants. The second condition is independence, where the instrumental variables are not related to error terms that affect the health of older migrants. We referred to studies by Tabllini (45), and selected a policy “Whether communities implement basic public health service projects (Yes = 1, No = 0)” as the instrumental variable. On one hand, community health services are part of this project, and its implementation directly affects the access of health education and health records for older migrants. On the other hand, as a public health policy, this project is not related to the health status of older migrants and belong to exogenous variable.

The regression results of the instrumental variables were reported in Table 5. Firstly, the stage I regression results reported the effects of instrumental variable on community-based health services. The national basic public health service project has a significant positive impact on the community-based health education. The implementation of this project will increase the probability of getting the community health education and health records increased by 45.1 and 45.4%, respectively. Secondly, the stage II regression results reported the effect of endogenous explanatory variable on the health of older migrants. Community health education still had a positive impact on the self-rated health of the older migrants after the inclusion of instrumental variables, with a significant correlation at the level of 1%. However, the effect of community health records on the self-rated health of the older migrants was not statistically significant. After dealing with the endogenous problem of community health services, the effect of community health services on the self-rated health of the older migrants remains robust.

**Table 5:** Instrumental variable estimation results (*N* = 5,340).

**Variables**	**Model 7**		**Model 8**	
	**Stage I**	**Stage II**	**Stage I**	**Stage II**
National basic public health service project	0.451^***^ (0.080)		0.454^***^ (0.011)	
Community-based health education		0.013^***^ (0.003)		
Community-based health records				−0.068 (0.046)
Control variables	Controlled		Controlled	
P (Durbin-Wu-Hausman Test)	0.002		0.011	
F (weak instrumental variable test)	22.51		20.19	

^***^Denote the significance of 1%, standard errors are contained within parentheses. In model 7, the endogenous explanatory variable is community-based health education, the dependent variable in the first stage is the health of older migrants, the independent variable is national basic public health service project, the dependent variable in the second stage is community-based health education, and the independent variable is the health of older migrants; In model 8, the endogenous explanatory variable is community-based health records, the dependent variable in the first stage is the health of older migrants, the independent variable is national basic public health service project, the dependent variable in the second stage is community-based health records, and the independent variable is the health of older migrants.

Besides, we examined several conditions that the instrumental variable needs to satisfy. Firstly, we examined whether the explanatory variables were endogenous variables. The *P*-values obtained through the DWH test in models 7 and 8 are 0.002 and 0.011, respectively, both of which are < 0.05. Therefore, community health education and community health records can be considered as endogenous variables. Secondly, we examined whether there was a weak instrumental variable problem. The F-value of the weak instrumental variable test in models 7 and 8 are 22.51 and 20.19, respectively, both >10. Therefore, it can be considered that there was no risk of weak instrumental variables.

Finally, we examined whether the instrumental variable was an exogenous variable. The Hausman test cannot be conducted since only one instrumental variable was selected in this paper. Therefore, we followed Fang's approach to examine the externality of instrumental variables (46). The instrumental variable is considered exogenous if it influences the dependent variable through the endogenous explanatory variable, but cannot directly or indirectly influence the dependent variable through any other means (47). Table 6 reported the test results of the externality of instrumental variables. When endogenous explanatory variable was controlled, the effect of National basic public health service project on the health of older migrants was not significant. However, the National basic public health service project had a significant impact on the health of older migrants when regression was performed separately, indicating that the instrumental variables did not directly affect the dependent variables, but only affected the health of older migrants through community-based health education. Therefore, the instrumental variables selected for this paper are exogenous. The satisfaction of the aforementioned conditions indicates that choosing the National Basic Public Health Service Project as instrumental variables is reasonable, as it effectively addresses the endogenous problem of this paper.

**Table 6:** The test results of the externality of instrumental variables (*N* = 5,340).

**Variables**	**Model 9**	**Model 10**	**Model 11**
Community-based health education	0.034^***^ (0.008)		0.035^***^ (0.009)
National basic public health service project		0.053^***^ (0.008)	0.028 (0.058)
Control variables	Controlled	Controlled	Controlled

^***^Denote the significance of 1%, standard errors are contained within parentheses. In model 9, the independent variable is community-based health education, the dependent variable in the health of older migrants; In model 10, the independent variable is national basic public health service project, the dependent variable is the health of older migrants; In model 11, the independent variable are community-based health education and national basic public health service project, the independent variable is the health of older migrants.

## 4 Discussion

This research is the first community-based perspective to study the health of Chinese older migrants. The study has analyzed the correlation between community-based health services and the health of older migrants, through nationally representative data. This study has also comprehensively explored the impact mechanisms and transmission pathways involved in this relationship. The self-rated health status of older migrants has proved to positively associate with community-based health education, not only in a direct pathway but also occur through social integration of older migrants. However, there were no positive relationships between community-based health records and older migrants' health. The findings of this research have indicated that community-based health services and social integration are crucial elements in improving the self-perceived health status of older migrants.

Firstly, this research revealed that community-based health education had a direct impact on the health of older migrants. The greater variety of health education provided by the community along with the establishment of community health records promoted self-related health among older migrants. This is consistent with previous research that health resource allocation and health promotion activities increased residents' utilization of community health services, which enhances their health (48, 49). A reasonable explanation is the feasible capability theory, empowering competence plays an imperative function in enhancing individual health (50). For the older migrant population, community-based health services are both a community resource and an important form of cultivating health literacy among vulnerable groups of older migrants (51). Communities utilize various forms as health education lectures, publicity brochure and internet websites, to provide health education content that covers chronic ailment prevention and physiological wellbeing. Such initiatives not only directly improve the knowledge of health of older migrants, but also enhance their capability for self-preventive care and disease prevention, thereby improving their overall health status (15).

Secondly, the community health records showed a negative correlation with the health of the older migrants, although this correlation was not statistically significant. The negative coefficient between community health records and the health of older adult immigrants is related to the supply pattern of public health services in China. For a long time, the provision of public services such as medical care and older adult care in China has been tied to household registration, resulting in exclusion of the older migrants from the coverage of basic public services (52). When older migrants find that local residents or those with rural hukou have a “comparative advantage” in accessing local public medical services, their self-rated health levels will decline. This is essentially consistent with existing research (12, 37), reflecting the negative impact caused by the reform of China's household registration system. However, the lack of statistically significant association between community health records and the health of older migrants might be attributed to the overall low establishment of health records among the respondents. This study discovered the only 33% of older migrants having community health record, and far from the target and requirement of 80% of the construction rate of the migrant population in China (53). Approximately 70% of older migrants have not established community health records, and the comparative advantages derived from accessing public services are not evident. At the same time, previous studies have also shown that the construction of health records for older migrants generally has the problem of low utilization rate and excessive form (54), which may make it difficult for health records to be transformed into practical abilities such as timely identify health risk factors, cope with chronic illnesses, or minimize the incidence of diseases, and consequently have limited influence on older immigrants' health. This indicates that there is a need for more government work on achieving the goal of equalizing public health services for the older migrants in China, and community platforms should be utilized to gain a fully understanding of their health situations, promote the establishment of health records, enhance their health awareness and provide timely healthcare services.

Thirdly, an important finding of this paper is that social integration plays a mediating role in the relationship between community-based health services and older migrants health. On the one hand, community-based health education is positively associated with social integration of older migrants. For the older migrants, constraints of household registration system make it difficult for them to develop an identity and social integration (55). Relying on older migrants' communities to create opportunities for them to participate actives in a reasonable and formal manner, which is a positive and effective approach for accelerating social integration (56). In the process of providing health education, actually the community also creates a platform for the older migrants to interact with local residents, and the establishment of local social network, which promotes them to develop a sense of self-identity that “I am a local”. More importantly, the community-based health education enables the older migrants to receive the same health service resources as the locals, which can reduce the inequity caused by the urban-rural dichotomy (57), and eliminate the integration obstacle that “locals look down on foreigners”, thus increasing the social integration of the older migrants.

On the other hand, social integration was positively associated with the health of older migrants. Based on the opportunities and platforms created by the community, the sense of belonging and willingness to socially integrate generated by the floating population can promote their self-rated health. This was an explanation based on social capital theory, where the degree of social integration reflects the local social network and social interaction status of the older migrants, which constitute the social capital of the older migrants in the community. The positive relationship between social capital and health has been studied and argued by numerous scholars (34, 58). Social capital can positively influence health by intervening in social networks to meet the health needs of the older migrants. The mediating effect implies the significance of providing community-based health services for the older migrants, leveraging the service and platform capabilities of the community, which can help mitigate barriers to social integration of the older migrants and assist them in establishing formal opportunities and pathways for social integration, thereby fostering toward their health and wellbeing in a comprehensive approach.

However, this study has several limitations. Firstly, due to data limitations, this study mainly used self-rated health and disease status indicators to evaluate the health of older migrants, further research should to provide a more comprehensive assessment of their overall health. Secondly, when measuring community-based health service indicators, the measurement of community health education was only examined in terms of service content, the form and frequency of community health education were not explored. Thirdly, this study only discussed the mediating mechanism of social integration in the relationship between community-based health services and the health of older migrants, and other potential mechanisms such as physiological or psychological pathways were not explored. Therefore, caution should be exercised when interpreting the representativeness of the study findings. In order to promote active aging and healthy aging among the older migrant population, more contents and forms of community-based health services need to be explored in the future.

## 5 Conclusion

Relying on a national sample of Chinese older migrants, this study focused on how the community-based health services may influence the health of older migrant. This study found a significant association of community-based health education with higher self-rated health among older migrants, and social integration played a mediating role in the positive association. However, the findings failed to support the favorable function of community health records in older migrants' health. These findings underscore the critical position of the community-based health services in improving the health of older migrants. The “Healthy China” strategy is a long-term plan implemented by the Chinese government to promote national health and enhance the overall wellbeing of the population. Given the vulnerability of their health and the complexity of migration, prioritizing older migrants' health is essential for achieving the goals, such as developing policies and regulations to support the health of older migrants, establishing a community-based health management platform.

Adopting a community perspective toward older migrants not only benefits their wellbeing, but also facilitates the basic public service equalization. It is therefore crucial to explore community-based interventions that establish convenient health service points in the community. These interventions should include regular health check-ups, various health education programs, disease screening activities, chronic disease management and emergency medical services to ensure that older migrants have easy access to quality healthcare. Meanwhile, fostering social support networks can contribute to the overall wellbeing and integration of older adult individuals into their community. This can be achieved through setting up clubs and activity centers, creating platforms for older migrants to socialize, and organizing cultural events such as traditional festivals and exhibitions. The results of this study provide guidance for decision-makers to implement personalized health education programs for older migrants, covering chronic diseases and mental health. It also suggests providing social support strategies such as social activities and neighborhood assistance. In addition, future research should include exploring specific aspects of community-based health services or delving into the effectiveness of different intervention strategies.

## Data availability statement

Publicly available datasets were analyzed in this study. This data can be found here: https://www.chinaldrk.org.cn/wjw/#/data/classify/population/yearList.

## Ethics statement

Ethical approval was not required for the study involving humans in accordance with the local legislation and institutional requirements. Written informed consent to participate in this study was not required from the participants or the participants' legal guardians/next of kin in accordance with the national legislation and the institutional requirements.

## Author contributions

SL: Conceptualization, Data curation, Formal analysis, Methodology, Project administration, Software, Validation, Writing – original draft. BQ: Investigation, Methodology, Project administration, Supervision, Writing – review & editing. DW: Conceptualization, Funding acquisition, Investigation, Methodology, Project administration, Resources, Supervision, Writing – review & editing.
